# Metabolic signatures of muscle mass loss in an elderly Taiwanese population

**DOI:** 10.18632/aging.202209

**Published:** 2020-12-19

**Authors:** Chi-Jen Lo, Yu-Shien Ko, Su-Wei Chang, Hsiang-Yu Tang, Cheng-Yu Huang, Yu-Chen Huang, Hung-Yao Ho, Chih-Ming Lin, Mei-Ling Cheng

**Affiliations:** 1 Metabolomics Core Laboratory, Healthy Aging Research Center, Chang Gung University, Taoyuan 333, Taiwan; 2 Division of Cardiology, Chang Gung Memorial Hospital, Taipei 105, Taiwan; 3 College of Medicine, Chang Gung University, Taoyuan 333, Taiwan; 4 Clinical Informatics and Medical Statistics Research Center, Chang Gung University, Taoyuan 333, Taiwan; 5 Department of Thoracic Medicine, Chang Gung Memorial Hospital, Taoyuan 333, Taiwan; 6 Graduate Institute of Biomedical Sciences, College of Medicine, Chang Gung University, Taoyuan 333, Taiwan; 7 Clinical Metabolomics Core Laboratory, Chang Gung Memorial Hospital, Taoyuan 333, Taiwan; 8 Division of Internal Medicine, Chang Gung Memorial Hospital, Taipei 105, Taiwan; 9 Department of Health Management, Chang Gung Health and Culture Village, Taoyuan 333, Taiwan; 10 Department of Biomedical Sciences, College of Medicine, Chang Gung University, Taoyuan 333, Taiwan

**Keywords:** metabolomics, sarcopenia, muscle mass loss, amino acid-related metabolites, elderly

## Abstract

To identify the association between metabolites and muscle mass in 305 elderly Taiwanese subjects, we conducted a multivariate analysis of 153 plasma samples. Based on appendicular skeletal muscle mass index (ASMI) quartiles, female and male participants were divided into four groups. Quartile 4 (Men: 5.67±0.35, Women: 4.70±0.32 Kg/m^2^) and quartile 1 (Men: 7.60±0.29, Women: 6.56±0.53 Kg/m^2^) represented low muscle mass and control groups, respectively. After multivariable adjustment, except for physical function, we found that blood urea nitrogen, creatinine, and age were associated with ASMI in men. However, only triglyceride level was related to ASMI in women. The multiple logistic regression models were used to analyze in each baseline characteristic and metabolite concentration. After the adjustment, we identify amino acid-related metabolites and show that glutamate levels in women and alpha-aminoadipate, Dopa, and citrulline/ornithine levels in men are gender-specific metabolic signatures of muscle mass loss.

## INTRODUCTION

Sarcopenia is a geriatric syndrome characterized by progressive loss of muscle mass and strength, placing the elderly at increased risk of disability, falls, and frailty [1]. The International Working Group on Sarcopenia [2], the European Working Group on Sarcopenia in Older People, the Asian Working Group for Sarcopenia (AWGS), and the Foundation for the National Institutes of Health Sarcopenia Project [3] recommend muscle function (strength or performance) and muscle mass measurements for a sarcopenia diagnosis [4, 5].

Optimal methods for modeling handgrip strength in statistical prediction remain controversial [6]. Although it has been suggested that low muscle mass is a poor indicator of functional outcomes when compared with muscle strength and performance [7], low muscle mass is a key component of the sarcopenia phenotype [3]. Pre-sarcopenia is characterized by the presence of low muscle mass with normal muscle strength and physical performance [8].

Previous studies have identified metabolites associated with lean mass or body mass index (BMI) [9–11]. These studies also reported the association between metabolic profiles and body composition. Metabolomics employs technologies aimed at better understanding the complexity of a living system. In translational research, a metabolomics approach may enable the detection of multiple disease risk factors and interactions, disease progression, and responses of patients to a particular therapy with or without side effects [12]. Amino acid metabolic disturbances [13] and anomalous energy metabolism [14] have been reported in patients with chronic fatigue syndrome, whereas abnormalities in 20 metabolic pathways have also has been reported [15]. Limited information is available regarding the effects of age-related sarcopenia on plasma metabolite levels [10, 16]. Recently, metabolomic and lipidomic analyses have been used to investigate gender differences under physiological and pathological conditions [17–19]. Gender is considered one of the most relevant biological variables influencing metabolomic and lipidomic profiles [20]. However, the relationship between circulating metabolites and gender, specifically in older adults with muscle mass loss, has not yet been characterized.

This study explores the association between metabolites and muscle mass in a healthy, elderly Taiwanese population. The independent subjects enrolled in our study lived in a retired home, without nursing assistance, and their score of activities of daily living (ADL) and instrumental activities of daily living (IADL) were intact. Although gender differences are identified in the metabolic signatures of muscle mass loss, and these metabolites are associated with the urea cycle. The higher catabolic rate of amino acids is linked with muscle mass loss. From these results, we can identify the metabolic biomarkers for age-related muscle mass loss or sarcopenia. Amino acid signatures can also be used to evaluate the beneficial effects of intervention.

## RESULTS

### Enrollment of study participants

A total of 305 subjects were enrolled in our study; 173 women and 132 men were eligible for muscle function, composition, and other clinical parameters, including appendicular skeletal muscle mass, handgrip strength, and gait speed. The average age of all participants was 81.8 years old, and 56.7% of the subjects were women. The average age of the female and male participants was 80.15 ± 7.08 and 83.95 ± 6.78 years old, respectively.

### Association of muscle mass with demographic and clinical characteristics

**Figure 1:**
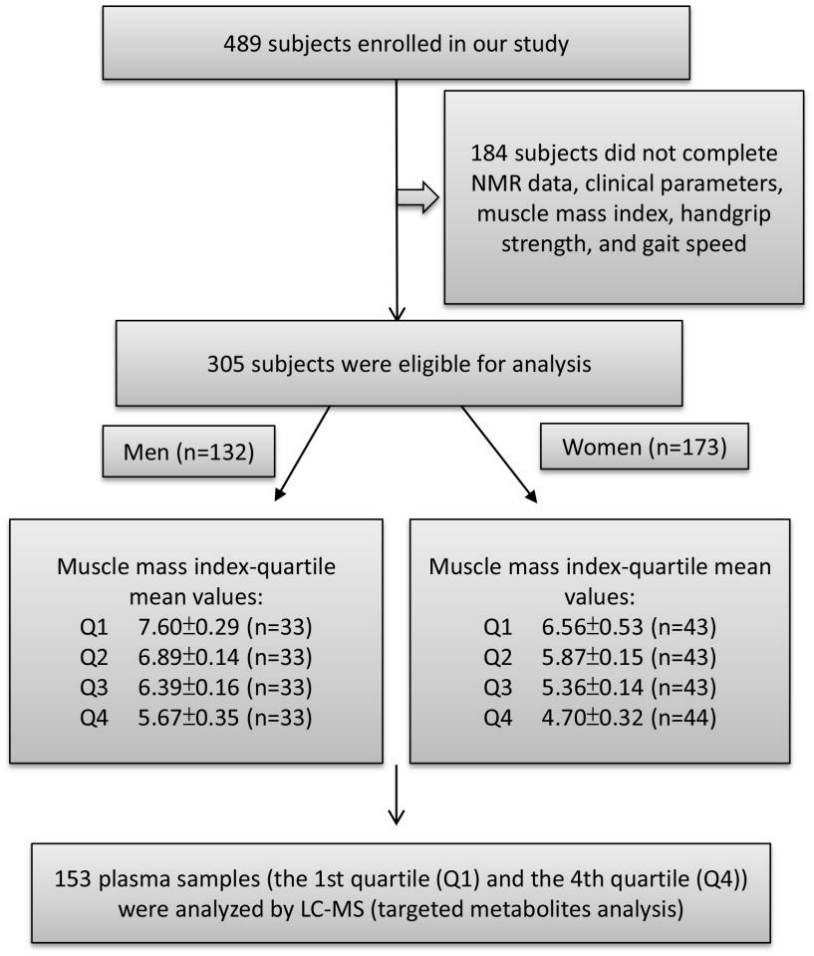
**Study flow diagram shows number of participants for untargeted and targeted metabolite analysis.** A total of 489 participants enrolled in this study of which 305 subjects were eligible to participate. According to appendicular skeletal muscle mass index (ASMI) values, we divided the male and female subjects into four groups each by quartile. The ASMI values of quartile 1, 2, 3, and 4 (Q1, Q2, Q3, and Q4) were 7.60±0.29, 6.89±0.14, 6.39±0.16, and 5.67±0.35 kg/m^2^, respectively. In the female group, quartile 1, 2, 3, and 4 (Q1, Q2, Q3, and Q4) ASMI values were: 6.56±0.53, 5.87±0.15, 5.36±0.14, and 4.70±0.32 kg/m^2^, respectively. The first quartile (Q1) was defined as the control group and the fourth quartile (Q4) as the muscle loss group. Both Q1 and Q4 were performed for metabolite analysis.

Female and male participants were divided into four groups each, based on their quartile, according to their appendicular skeletal muscle mass index (ASMI) values (appendicular skeletal muscle mass divided by height squared, kg/m^2^). ASMI cutoff values were: quartile 1 (Q1): > 7.153 kg/m^2^; quartile 2 (Q2): 6.659–7.128 kg/m^2^; quartile 3 (Q3): 6.155–6.658 kg/m^2^; and quartile 4 (Q4): < 6.155 kg/m^2^ in male participants, and Q1: > 6.125 kg/m^2^; Q2: 5.625–6.114 kg/m^2^; Q3: 5.149–5.604 kg/m^2^; and Q4: < 5.149 kg/m^2^ in female participants (Figure 1). Specifically, we used the sarcopenia cutoff values recommended by the AWGS for loss of muscle mass (ASMI: < 7.0 kg/m^2^ for men and < 5.4 kg/m^2^ for women) and loss of muscle function (handgrip strength: < 26 kg for men and < 18 kg for women; gait speed: < 0.8 m/s). According to AWGS criteria, participants in Q1 present normal ASMI values (> 7.153 kg/m^2^ for men and > 6.125 kg/m^2^ for women), however, participants in Q4 present muscle mass loss (< 6.155 kg/m^2^ for men and < 5.149 kg/m^2^ for women). ASMI Q1, Q2, Q3, and Q4 values were 7.60 ± 0.29, 6.89 ± 0.14, 6.39 ± 0.16, and 5.67 ± 0.35 kg/m^2^, respectively, in male participants, and 6.56 ± 0.53, 5.87 ± 0.15, 5.36 ± 0.14, and 4.70 ± 0.32 kg/m^2^, respectively, in female participants. Table 1 shows the proportion of loss of muscle mass and function in male and female participants in different quartiles. Higher muscle mass loss was observed in Q4 compared with that in Q1 (Table 1). In both male and female groups, handgrip strength declined in Q4 compared with that in Q1. However, in the female group, the gait speed of Q4 was not significantly lower than that of Q1 (Table 1).

**Table 1:** Baseline demographics of men and women in Taiwanese elderly population.

**Quartile group**	**Men**				**Women**			
	**Q1**	**Q4**	**P value**	**Adj P_FDR_**	**Q1**	**Q4**	**P value**	**Adj P_FDR_**
**characteristics**	**(N=33)**	**(N=33)**	**(Q1 vs. Q4)**	**(Q1 vs. Q4)**	**(N=43)**	**(N=44)**	**(Q1 vs. Q4)**	**(Q1 vs. Q4)**
Age	82	87	0.0011	0.0319	81	81.5	0.7790	
Muscle mass and strength								
Walking speed, m/sec	1.20	0.91	0.0076	0.0005	1	1.04	0.3395	0.0065
Grip strength, kg	25.5	21	0.0018	0.0034	15.9	13.15	0.0261	0.0129
ASMI, Kg/m2	7.60	5.77	3.02E-12	9.86E-25	6.44	4.76	9.98E-16	4.95E-26
Body mass								
Height, cm	165.7	164.1	0.7291		153.6	152.2	0.8022	
Weight, kg	71.9	55.8	8.17E-07	2.20E-05	62.8	46.65	2.81E-14	1.18E-13
BMI, kg/m2	25.72	20.91	2.09E-08	2.75E-07	26.38	19.09	4.50E-15	7.45E-18
waist, cm	93	82	0.0003	0.0032	93	82	2.75E-09	4.19E-06
Blood pressure, mmHg								
Systolic	128	126	0.9081		127	128.5	0.7309	
Diastolic	69	67	0.4640		69	66	0.0171	
Laboratory data								
WBC, 103/ml	5.5	5.3	0.7975		5.3	5.35	0.8617	
RBC, 106/ml	4.46	4.16	0.0307	0.5276	4.46	4.12	0.0686	
Hb, g/dl	14.2	12.6	0.0008	0.2866	13.5	12.6	0.0313	
Platelets, 103/ml	179	193	0.4686		210	227	0.4078	
Cholesterol, mg/dL	166	175	0.3393		177	183	0.9831	
Triglyceride, mg/dL	91	78	0.0856		92	84.5	0.0360	0.0499
HDL-C, mg/dL	49	50	0.6533		51	58.5	0.0122	0.2555
LDL-C, mg/dL	96	105	0.2899		106	103	0.5898	
Glucose, mg/dL	96	95	0.8928		100	90.5	0.0030	
HbA1c, %	5.8	5.7	0.5671		5.75	5.8	0.2222	
Uric acid, mg/dL	5.6	6.2	0.0786	0.1272	5.3	4.75	0.0870	
Albumin, g/dL	4.32	4.19	0.0054	0.4556	4.41	4.375	0.8219	
Total protein, g/dL	7	7	0.6244		7	7.2	0.1811	
AST/GOT, U/L	25	23	0.0870		26	27	0.6552	
ALT/GPT, U/L	17	13	0.0072		18	15	0.0116	
ALKP, U/L	69	59	0.0551	0.1838	63	61.5	0.5077	
Total bilirubin, mg/dL	0.9	0.7	0.0147	0.5612	0.7	0.6	0.0406	0.2792
BUN, mg/dL	16.8	22.8	0.0003	0.0027	15.1	15.35	0.7696	
Creatinine, mg/dL	0.93	1.05	0.0993	0.0113	0.67	0.65	0.1585	
Na, mEq/L	142	142	0.3392		143	142.5	0.2223	
K, mEq/L	4.2	4.3	0.1747		4.1	4.25	0.8015	
Cl, mEq/L	105	105	0.7074		106	106	0.2949	
Ca, mg/dL	9.1	9	0.3438		9.2	9.2	0.9456	
Comorbidity			Chi-square test P				Chi-square test P	
Hypertension (%)	61%	55%	0.6184		63%	43%	0.0670	
Diabetes (%)	24%	30%	0.5804		33%	23%	0.3050	
Hyperlipidemia (%)	27%	30%	0.7857		42%	25%	0.0953	
CAD (%)	6%	12%	0.3918		14%	5%	0.1289	
Cancer (%)	9%	9%	1.0000		2%	5%	0.5705	
Stroke (%)	12%	12%	1.0000		14%	0%	0.0102	
CKD (%)	9%	21%	0.1697		9%	7%	0.6702	
COPD (%)	24%	42%	0.1178		9%	20%	0.1446	
Osteoporosis (%)	9%	24%	0.0986		33%	57%	0.0229	

* Data are medians. Variables were analyzed by Mann-Whitney U tests. Multiple logistic regression models were used to analyze the effect of gender on the dependent variable controlling for age and comorbidities, including hypertension, diabetes, hyperlipidemia, coronary artery disease (CAD), cancer, stroke, chronic kidney disease (CKD), chronic obstructive pulmonary disease (COPD), and osteoporosis. Model significance was presented in adjusted P value. ASMI, appendicular skeletal muscle mass index; BMI, body mass index; WBC, white blood cell; RBC, red blood cell; Hb, hemoglobin; HDL-C, high-density lipoprotein-cholesterol; LDL-C, low density lipoprotein-cholesterol; HbAlc, hemoglobin A1c; AST, aspartate aminotransferase; ALT, alanine aminotransferase; ALKP, alkaline phosphatase; BUN, blood urea nitrogen.

After multivariable adjustment, except for physical function, body composition parameters, including gait speed, handgrip strength, weight, BMI, waist circumference, age, and other parameters, such as blood urea nitrogen (BUN) and serum creatinine, were associated with ASMI in men; however, only triglyceride was related to ASMI in women. The proportion of subjects with osteoporosis was higher in Q4 than in Q1 in the female group (Table 1). Therefore, our results suggested that the clinical parameters associated with muscle mass loss were gender-specific in elderly Taiwanese population.

### Association of muscle mass with metabolite profiles

**Figure 2:**
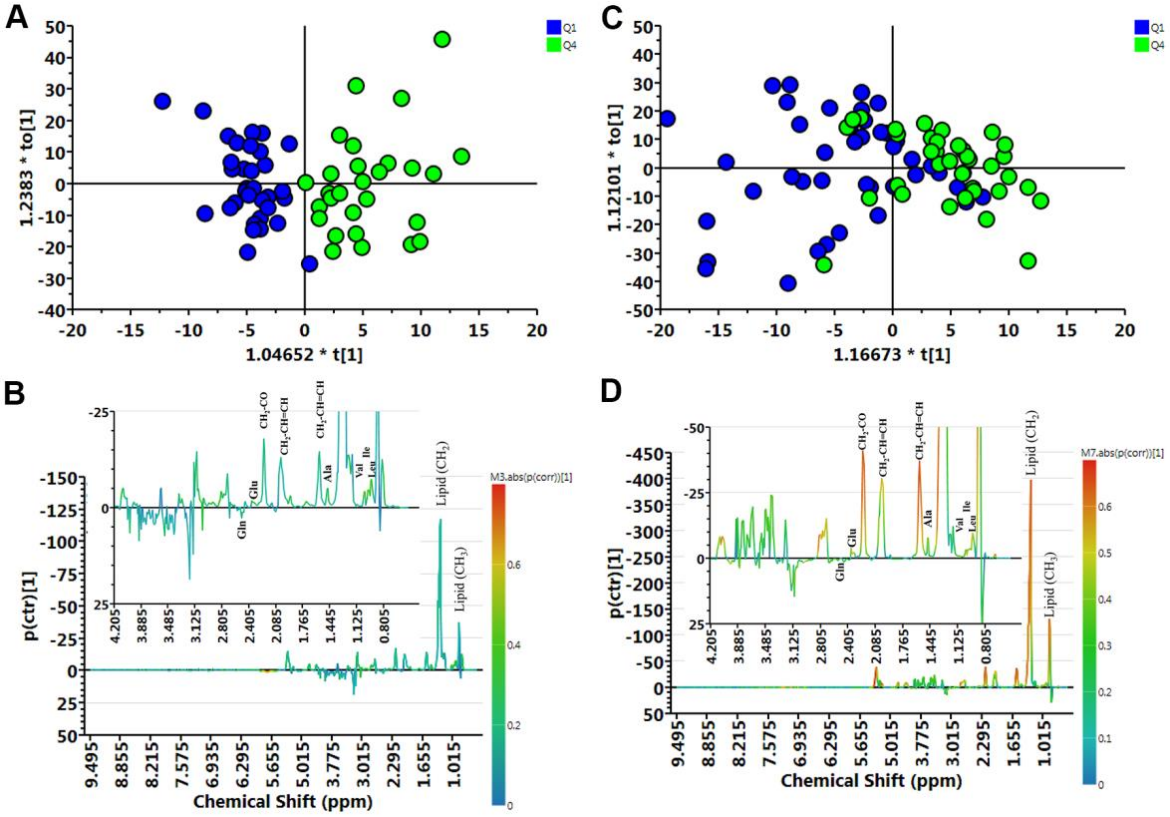
**Muscle loss and control group samples analyzed by ^1^H NMR spectroscopy.** (**A**) Orthogonal partial least-squares discriminant analysis plot (OPLS-DA) of the control (Q1) and muscle loss (Q4) subgroups in men showed considerable separation (R2X = 0.404, R2Y = 0.750, and Q2 = 0.316). (**B**) Contribution plots of control (Q1) and muscle loss (Q4). (**C**) OPLS-DA plot of control (Q1) and muscle loss (Q4) subgroups in women showed considerable separation (R2X = 0.362, R2Y = 0.360, and Q2 = 0.00307). (**D**) Contribution plots of control (Q1) and muscle loss (Q4). The results were revealing amino acid-related metabolites as important discriminators between Q1 and Q4.

We compared the metabolite profile of plasma between normal (Q1) and muscle mass loss (Q4) groups in elderly Taiwanese subjects by untargeted nuclear magnetic resonance (NMR) analysis of 153 plasma samples. The metabolites contributing to the distinction between Q1 and Q4, in both female and male participants, are shown in distribution plots, revealing amino acid-related metabolites as important discriminators between Q1 and Q4 (Figure 2).

### Metabolites associated with muscle mass loss

Quantification of amino and biogenic amines was performed by liquid chromatography-mass spectrometry (LC-MS), and the results are shown in Table 2. In the female group, the levels of total amino acids, including essential and nonessential amino acids, decreased and significantly changed in Q4. In the male group, essential amino acids, aromatic amino acids (AAAs), branched amino acids (BCAAs), glutamate, aspartate, tryptophan, threonine, alpha-aminoadipate (alpha-AAA), and sarcosine levels decreased in Q4, whereas the levels of biogenic amines, such as symmetric dimethylarginine (SDMA), Dopa, kynurenine/tryptophan, citrulline/ornithine (Cit/Orn), and putrescine/Orn ratios increased in Q4 compared with those in Q1.

**Table 2:** Concentration of metabolites significantly differentially expressed between Q1 and Q4 groups.

	**Men**			**Women**		
**Amino acid (μM)**	**Q1**	**Q4**	**P**	**Q1**	**Q4**	**P**
	**(N=33)**	**(N=33)**	**(Q1 vs. Q4)**	**(N=43)**	**(N=44)**	**(Q1 vs. Q4)**
Essential_AA	1121.6	1056	0.0402	1006.4	916.4	0.0037
Ile	82	75.7	0.0929	69.4	62.9	0.0219
Leu	153	136	0.0085	131	116.5	0.0033
Met	29	27.5	0.5682	26.2	23.45	0.0276
Phe	77.6	71.7	0.0980	70.7	65.15	0.0253
Thr	141	121	0.0202	118	107.5	0.1104
Val	261	230	0.0183	248	217	0.0018
Non_essential_AA	2219.48	2252.27	0.8878	2200.86	2057.44	0.0023
Ala	387	369	0.5813	395	341.5	0.0028
Asp	2.37	1.59	0.0236	2.2	1.515	0.0324
Glu	49	44.5	0.1930	65.2	41.25	2.90E-05
His	100	94.5	0.1074	97.2	90.45	0.0073
Pro	170	169	0.8524	151	134	0.0069
Trp	59.2	54.1	0.0141	56.7	53.9	0.1040
Total_AA	3332.09	3311.84	0.4727	3221.82	3020.73	0.0014
BCAA	496.7	442	0.0236	456.6	393.15	0.0027
AAA	215	200	0.0257	206.9	189.05	0.0200
Gln/Glu	14.70	18.01	0.2088	10.46	17.56	0.0001
Cit/Orn	0.31	0.35	0.0031	0.30	0.35	0.0082
Orn/Arg	1.73	1.35	0.0133	1.92	1.65	0.0288
Kynurenine/Trp	0.04	0.05	0.0063	0.04	0.04	0.9560
Putrescine/Orn	1.15E-03	1.36E-03	0.0667	9.71E-04	1.30E-03	0.0841
Glucogenic AA	779	760.7	0.7004	786	749.4	0.0288
DOPA	0.16	0.18	0.0736	0.18	0.17	0.7277
Sarcosine	8.61	6.72	0.0037	7.56	7.40	0.8485
SDMA	0.75	0.94	0.0052	0.69	0.65	0.4576
alpha_AAA	1.03	0.82	0.0131	0.93	0.72	0.0017
ADMA/Arg	0.01	0.01	0.7388	0.01	0.01	0.0157

Data are medians. Variables were analyzed by Mann-Whitney U tests.

Alpha-AAA, Dopa, Cit/Orn ratio, and age were significantly associated with muscle mass loss after multivariable stepwise adjustment in men (Table 3A); however, only glutamate was significantly associated with muscle mass loss in women (Table 3B). After multivariable stepwise adjustment, glutamate and Cit/Orn ratio were significantly associated with muscle mass loss in all subjects (Table 3C). These metabolites are involved in the urea cycle. It is likely that a higher catabolic rate of amino acids is linked with muscle mass loss in elderly Taiwanese subjects.

**Table 3A:** Multivariable analyses of muscle mass and metabolite associations in men.

**Male**	**Q1**	**Q4**	**P**	**Parameter Estimate (SE) Before Adjustment**	**Parameter Estimate (SE) After Adjustment**	**Multivariable -Stepwise**
	**(N=33)**	**(N=33)**	**(Q1 vs Q4)**			**P-value**
Age, yrs	80.52±7.83	87.03±4.22	0.0374	0.1797 (0.0556)	0.2828 (0.0998)	0.0046
Glu, μM	55.59±25.93	45.27±13.77	0.049	-0.0259 (0.0136)	NA	
Cit/Orn	0.30±0.08	0.39±0.12	0.0011	11.3751 (4.0146)	18.0713 (7.0956)	0.0109
DOPA, μM	0.15±0.06	0.18±0.05	0.0372	11.0399 (5.5216)	22.1837 (9.1725)	0.0156
alpha_AAA, μM	1.03±0.40	0.82±0.21	0.0087	-2.5589 (1.0321)	-3.1661 (1.4556)	0.0296

**Table 3B:** Multivariable analyses of muscle mass and metabolite associations in women.

**Female**	**Q1**	**Q4**	**P**	**Parameter Estimate (SE) Before Adjustment**	**Parameter Estimate (SE) After Adjustment**	**Multivariable -Stepwise**
	**(N=43)**	**(N=44)**	**(Q1 vs Q4)**			**P-value**
Age, yrs	80.02±8.14	80.23±6.95	0.5132	0.0037 (0.0287)	NA	
Glu, μM	67.80±31.00	40.88±14.30	2.84E-06	-0.0524 (0.0133)	-0.0524 (0.0133)	7.79E-05
Cit/Orn	0.29±0.10	0.35±0.11	0.0112	5.4286 (2.2554)	NA	
DOPA, μM	0.17±0.04	0.17±0.04	0.4844	-3.8584 (5.4678)	NA	
alpha_AAA, μM	0.95±0.28	0.75±0.26	0.0009	-2.7284 (0.8838)	NA	

**Table 3C:** Multivariable analyses of muscle mass and metabolite associations in all participants.

**All**	**Q1**	**Q4**	**P**	**Parameter Estimate (SE) Before Adjustment**	**Parameter Estimate (SE) After Adjustment**	**Multivariable –Stepwise**
	**(N=76)**	**(N=77)**	**(Q1 vs Q4)**			**P-value**
Age, yrs	80.24±7.96	83.14±6.81	0.3969	0.0532 (0.0225)	NA	
Glu, μM	62.49±29.36	42.76±14.15	6.65E-07	-0.0413 (0.0093)	-0.0440 (0.0102)	4.07E-05
Cit/Orn	0.29±0.09	0.37±0.12	4.82E-05	7.1674 (1.9484)	6.7456 (2.1960)	0.0021
DOPA, μM	0.17±0.05	0.17±0.04	0.2747	3.9774 (3.6411)	9.8519 (4.6787)	0.0352
alpha_AAA, μM	0.99±0.34	0.78±0.24	2.56E-05	-2.6296 (0.6673)	NA	

Data are mean ± SD. SE: Standard Error. Variables were analyzed by independent samples t-tests or Chi-square test (age). Multiple logistic regression models were used to analyze the effect of gender on the dependent variable controlling for age and comorbidities, including hypertension, diabetes, hyperlipidemia, coronary artery disease (CAD), cancer, stroke, chronic kidney disease (CKD), chronic obstructive pulmonary disease (COPD), and osteoporosis. Model significance was presented in adjusted P-value.

## DISCUSSION

**Figure 3:**
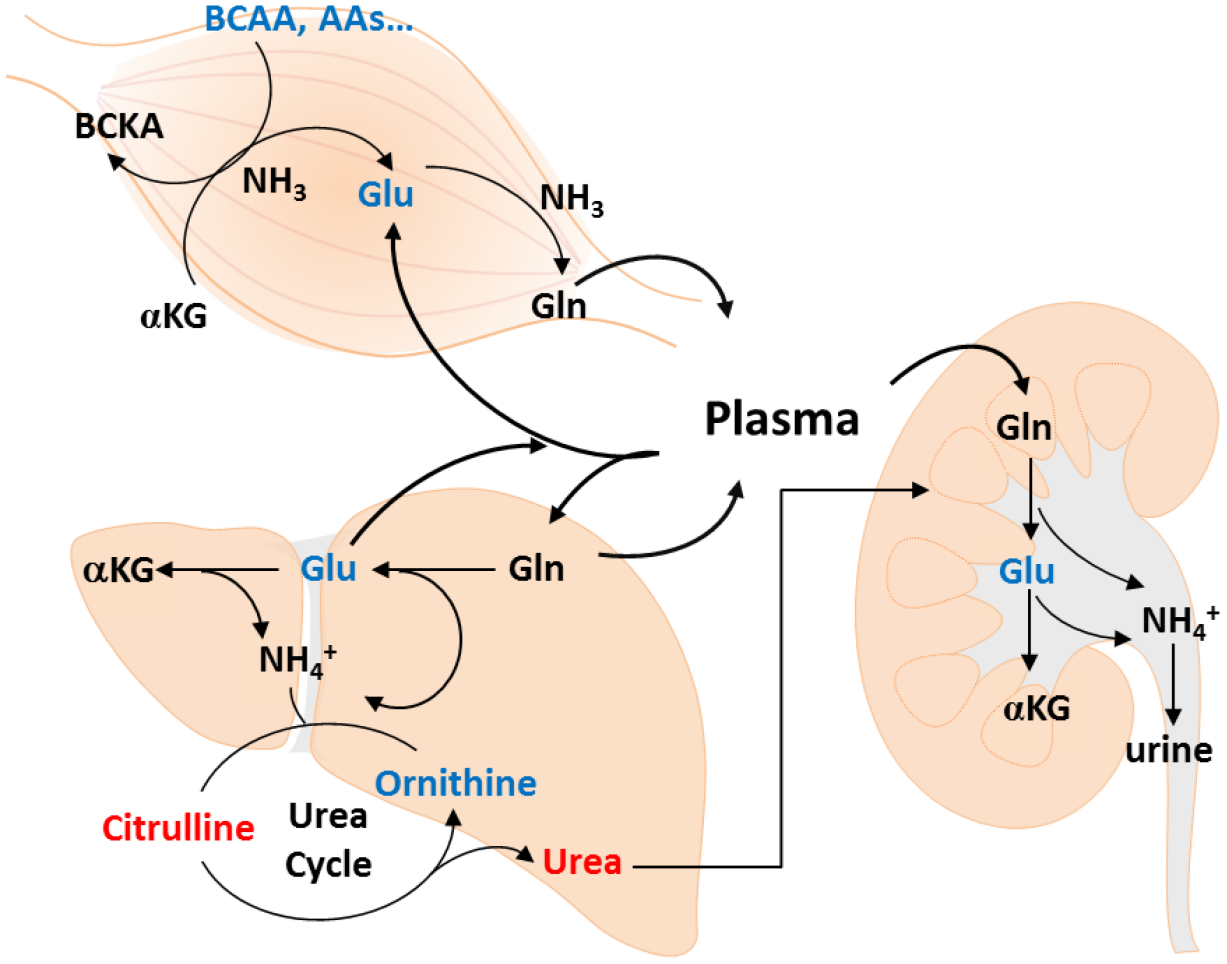
**Metabolic changes of muscle loss in Taiwanese elderly population.** Metabolic changes are mapped to pathways involved in amino acid catabolism, urea cycle, ornithine-proline-glutamate pathways, transamination, and glutaminolysis.

Some studies have analyzed age-related metabolite changes in humans [21], but rarely investigated gender-specific differences. Because gender has a great impact on plasma metabolic profiling [18], we used an NMR- and LC-MS-based metabolomics approach to detect muscle mass-associated plasma metabolites in each gender. Metabolite concentration, including essential amino acids, BCAA, AAA, glutamate, aspartate, as well as Cit/Orn and Orn/arginine ratios showed similar changes in both gender with muscle loss. Multivariate analyses indicated a gender-specific metabolite signature. The changes in Alpha-AAA, Dopa, and Cit/Orn ratio were associated with muscle mass loss in men, whereas the change in only glutamate was significantly associated with muscle mass loss in women. Evaluating the level of urea cycle-related metabolites, such as glutamate and Cit/Orn ratio may help to explore the possible role of muscle mass loss in the elderly population. The metabolite pathways, including the urea cycle, Orn-proline-glutamate pathways, transamination, and glutaminolysis, are shown in Figure 3.

An increased glutamine/glutamate ratio in the Q4 female group indicated accelerated breakdown of amino acids and insufficient glutamine anaplerosis to restore glutamate. Glutamine-derived glutamate supports the levels of many amino acid pools in the cell through the action of aminotransferases; suppresses the amino acid-sensing kinase, general control nonderepressible 2; and inhibits the activating transcription factor 4 (ATF4). ATF4 is a key mediator of age-related muscle weakness and atrophy, including starvation, muscle disuse, and aging [22–24]. In addition to its role in transamination reactions, glutamate can be used to produce glutamine, used for extracellular matrix production [25]. Aspartate plays key role in both purine and pyrimidine biosynthesis to support cell division, and the biosynthesis relying on both glutamate flux through the tricarboxylic acid cycle and glutamate transamination [26].

Although it is still unclear how glutamine anaplerosis declines during muscle mass loss, a recent study indicated that glutamate significantly associates with muscle mass and strength in Caucasian women [27]. The reduction of mitochondrial glutaminase expression and suppression of glutamine anaplerosis in aging mesenchymal stem cells has also been reported [28].

Several studies have shown impaired mitochondrial function in aging muscle [29]. It is likely that restoration of impaired glutamate uptake in aged skeletal muscle may coordinately decrease mitochondrial function or impair amino acid delivery.

Citrulline, an intermediate of the urea cycle and an endogenous precursor of arginine, plays a role in regulating nitrogen homeostasis [30, 31]. In our study, Cit/Orn ratio increased in the Q4 male group, indicating high nitrogen load in the urea cycle during muscle mass loss. Both BUN and creatinine levels increased in the Q4 male group, suggesting that nitrogen overload results from catabolism in aging skeletal muscle. In addition, several reports have shown that Cit levels in human blood increase with age [32, 33]. Multivariate analysis demonstrated that age and Cit/Orn ratio are associated with muscle mass loss in men. However, no differences in liver function were observed between male and female groups. Further investigation on nitrogen homeostasis and amino acid catabolic pathways is needed to explain the higher Cit/Orn ratio in older men with muscle mass loss.

Because the muscle tissue is a major site for glutamine synthesis in the human body, glutamine can be replenished by six amino acids, including leucine, isoleucine, valine, asparagine, aspartate, and glutamate [34]. In addition, enhanced glutamine synthase activity was found in the skeletal muscle of aged rats, but their plasma level of glutamine remained unchanged [35]. It is apparent that normal plasma levels of glutamine are insufficient to meet increased demands under stress. In elderly Taiwanese subjects, there was no significant change in glutamine plasma levels; however, glutamine/total amino acids ratio was higher in the muscle mass loss group in both genders. This implies that the amino acids in skeletal muscle are stored in catabolic state. In aged individuals, nitrogen excretion is enhanced through increase urea excretion in men, and the inhibition of glutamate in women. Although previous data have demonstrated the role of glutamine in age-related loss of muscle mass, in this study, we demonstrated the potential role of glutamine in regulating nutritional state during aging.

This study has several limitations. First, the cohort size of participants was small, with or without muscle mass loss in different gender groups. Second, metabolic biomarkers were not confirmed by a validation study. A follow-up longitudinal study is ongoing, under way for data validation. Notably, our study suggests that amino acid-related metabolites may be used as indicators of nutritional status and as potential biomarkers for muscle mass loss or sarcopenia.

## MATERIALS AND METHODS

### Setting

The retired home currently has about 700 residents. The average age of the residents is 81.2 years old, and 61.2% of the residents are female. Most residents have the same living environment and share similar lifestyles, including dietary habits and exercise routines. They also receive medical care at the same medical institutions. Healthy, independent subjects enrolled in our study lived in this retired home, requiring no nursing assistance and aging above 65 years. The residents managed their daily living activities, including light housework, preparing meals, taking medications, shopping, using the telephone, and managing money, using other technologies, as well as socializing and organizing social events. All of the criteria should be tallied that the Short Portable Mental Screening Questionnaire (SPMSQ) with a score of 0 indicates no error and the score of activities of daily living (ADL) and instrumental activities of daily living (IADL) were intact. Our participants, although they may have chronic diseases and need medication, they are a group of healthy people who can take care of their daily lives without the assistance of others. Meeting the above conditions is considered “healthy elderly”.

### Ethics statement

The study protocol was approved by the Institutional Review Board of Chang Gung Memorial Hospital. Written informed consent was obtained from all subjects.

### Study design and participants

This study was performed in 2014 at a retirement home in Northern Taiwan. Plasma samples were obtained from participants for hematological, biochemical, and metabolomics studies. In addition, handgrip strength, gait speed, and muscle mass were also measured to identify the risk factors of sarcopenia.

### Muscle mass index

The muscle mass of each participant was measured using dual energy X-ray absorptiometry (GE Lunar iDXATM; GE Healthcare, Madison, WI, USA), and the ASMI was calculated as appendicular skeletal muscle mass divided by height squared (kg/m^2^) [4, 36].

### Gait speed

Each participant was asked to walk a distance of 4 m to measure his or her gait speed [4].

### Handgrip strength

A hand dynamometer (Jamar Plus+ Digital Hand Dynamometer; Sammons Preston, Bollingbrook, IL, USA) was used to evaluate handgrip strength of each participant’s dominant hand [4].

### NMR analysis of plasma samples

Plasma samples from the aged cohort (n=153) were obtained in EDTA tubes (BD Vacutainer, Franklin Lakes, NJ, USA). Each plasma sample (350 μL) was mixed with 350 μL of plasma buffer solution [75 mM Na_2_HPO_4_, 0.08% TSP 3-(trimethylsilyl) propionic-2,2,3,3-d4 acid, 2 mM NaN_3_, and 20% D_2_O], and 600 μL of the supernatant was then transferred to 5 mm NMR tubes for analysis.

^1^H NMR spectra were acquired on a Bruker Avance III HD 600 MHz NMR spectrometer at 310 K using a 5-mm inverse triple resonance CryoProbe (^1^H/^13^C/^15^N) (Bruker Biospin GmbH, Rheinstetten, Germany). The spectra were acquired by Carr-Purcell-Meiboom-Gill spin-echo pulse sequence with water suppression, a 4-s relaxation delay, and 80-ms T2 relaxation time. All NMR spectra were phased and baseline-corrected using Topspin software (version 3.2.2; Bruker Biospin GmbH, Rheinstetten, Germany), and then referenced to the chemical shift of ^1^H α-glucose at 5.23 ppm [37]. After processing, NMR spectra should reach the criterion of quality control that the line width at half-height to lactate resonance at 1.32 ppm is < 1.15 Hz.

Each ^1^H NMR spectrum from the plasma samples was segmented into equal widths (0.01 ppm), corresponding to regions 9.5-0.5 ppm, and the spectral data were normalized to the reference compound TSP by AMIX (version 3.9.14; Bruker Biospin GmbH, Rheinstetten, Germany). The resulting data sets were analyzed by SIMCA-P+ (version 13.0; Umetrics, Umea, Sweden). Resonant frequencies of each metabolite were acquired from the in-house library and Chenomx NMR Suite 7.1 (Chenomx, Edomonton, Canada).

### Amino acids and biogenic amines quantification by LC-MS

A total of 153 plasma samples were analyzed with a commercially available kit (AbsoluteIDQ p180 kit; Biocrates Life Sciences AG, Innsbruck, Austria) using an Acquity BEH C18 column (75 mm × 2.1 mm, particle size of 1.7 μm) in the ultra-pressure liquid chromatography system (Waters Corporation, Milford, MA, USA) coupled with multiple reaction monitoring on a triple-quadrupole mass spectrometer (Xevo TQS-MS; Waters Corporation, Milford USA) operating in the multiple reaction monitoring. Metabolite concentrations were calculated and expressed as micrometers [38].

### Statistical analysis

Study subjects were divided into four groups, Q1, Q2, Q3, and Q4, based on the quartiles of their appendicular skeletal muscle mass levels. The baseline characteristics and metabolite concentrations were presented as medians for continuous variables, and as counts (percentages) for categorical variables. Comparisons between male and female participants were carried out using the Mann-Whitney U test. Multiple logistic regression models were used to analyze the difference between men and women in each baseline characteristic and metabolite concentration when controlling for age and comorbidities, including hypertension, diabetes, hyperlipidemia, coronary artery disease, cancer, stroke, chronic kidney disease, chronic obstructive pulmonary disease, and osteoporosis. Collinearity diagnostics and the variance inflation factor of the variables were investigated to avoid multicollinearity. Corresponding model significance was presented as “Adjusted P value”. In univariate analysis of metabolites, the visual infusion phlebitis score of each was evaluated and compared between the Q1 and Q4 groups. In addition, the combined effect of significantly differentially expressed metabolites was examined using multiple regression models for men and women. The “Multivariate P value” was calculated after controlling for age and the nine previously mentioned comorbidity factors. To account for multiple testing, the Benjamini and Hochberg linear step-up method was performed [39], and false discovery rate adjusted P values were calculated using the MULTTEST procedure in SAS software (SAS Institute, Cary, NC, USA). A corrected P value less than 0.05 was considered statistically significant.
